# Crowdsourcing as a Screening Tool to Detect Clinical Features of Glaucomatous Optic Neuropathy from Digital Photography

**DOI:** 10.1371/journal.pone.0117401

**Published:** 2015-02-18

**Authors:** Danny Mitry, Tunde Peto, Shabina Hayat, Peter Blows, James Morgan, Kay-Tee Khaw, Paul J. Foster

**Affiliations:** 1 NIHR Biomedical Research Centre, Moorfields Eye Hospital and UCL Institute of Ophthalmology, London, United Kingdom; 2 Department of Public Health and Primary Care, University of Cambridge, Strangeways Research Laboratory, Cambridge, United Kingdom; 3 School of Optometry and Vision Sciences, Cardiff University, Cardiff, United Kingdom; 4 Department of Clinical Gerontology, Addenbrookes Hospital, University of Cambridge, Cambridge, United Kingdom; Univ Rochester Medical Ctr, UNITED STATES

## Abstract

**Aim:**

Crowdsourcing is the process of simplifying and outsourcing numerous tasks to many untrained individuals. Our aim was to assess the performance and repeatability of crowdsourcing in the classification of normal and glaucomatous discs from optic disc images.

**Methods:**

Optic disc images (N = 127) with pre-determined disease status were selected by consensus agreement from grading experts from a large cohort study. After reading brief illustrative instructions, we requested that knowledge workers (KWs) from a crowdsourcing platform (Amazon MTurk) classified each image as normal or abnormal. Each image was classified 20 times by different KWs. Two study designs were examined to assess the effect of varying KW experience and both study designs were conducted twice for consistency. Performance was assessed by comparing the sensitivity, specificity and area under the receiver operating characteristic curve (AUC).

**Results:**

Overall, 2,540 classifications were received in under 24 hours at minimal cost. The sensitivity ranged between 83–88% across both trials and study designs, however the specificity was poor, ranging between 35–43%. In trial 1, the highest AUC (95%CI) was 0.64(0.62–0.66) and in trial 2 it was 0.63(0.61–0.65). There were no significant differences between study design or trials conducted.

**Conclusions:**

Crowdsourcing represents a cost-effective method of image analysis which demonstrates good repeatability and a high sensitivity. Optimisation of variables such as reward schemes, mode of image presentation, expanded response options and incorporation of training modules should be examined to determine their effect on the accuracy and reliability of this technique in retinal image analysis.

## Introduction

Glaucoma is a neurodegenerative disease of the optic nerve, characterized by morphologic changes in the optic disc and the retinal nerve fiber layer with corresponding loss in visual field. Signs associated with glaucomatous optic nerve damage include progressive enlargement of the optic cup, focal notches in the neuroretinal rim, optic disc hemorrhages, nerve fiber layer defects, and parapapillary atrophy.[1] In the last decade, there has been considerable interest in developing a screening tool for glaucomatous optic neuropathy using either expert graded imaging or automated detection[2–4], however to date, no individual method can be recommended.[5]

Crowdsourcing, the process of outsourcing small simplified tasks to a large number of individuals, is a novel and cost-effective way of classifying medical images.[6] The largest commercial crowdsourcing provider is Amazon’s Mechanical Turk. (https://www.mturk.com/mturk/welcome) MTurk is an Internet-based platform that allows requesters to distribute small computer-based tasks to a large number of untrained workers.

Using the MTurk platform, our aim was to assess the sensitivity and specificity of crowdsourcing as a technique to detect typical signs of glaucomatous optic neuropathy from colour fundus photographs.

## Methods

Images were extracted and anonymised, with permission, from studies undertaken at the Moorfields Eye Hospital Reading Centre (MEHRC). The images have been graded normal/abnormal by fully trained Graders at MEHRC. These were then adjudicated by the clinical lead of the Reading Centre. Those taken from diabetic retinopathy screening and deemed to have glaucomatous discs were all verified in a clinical setting by a glaucoma consultant (PJF) at Moorfields Eye Hospital. Those with normal discs were graded by at least two senior graders; and only those images with100% agreement between the graders and adjudicated normal by the clinical lead were included in this current set.

In total 127 disc images were used. Abnormal images were designated as those with thinning or notching of the neuro-retinal rim or the presence of peri-papillary hemorrhages. Normal images were designated as an absence of any of these features. All images were anonymised and uploaded onto an ftp site for the study duration, to allow remote access.

We used the MTurk Web platform for anonymous workers to perform a classification task of the optic nerve images in our dataset. MTurk employs knowledge workers (KWs), who are untrained individuals to carry out simple tasks. KWs are registered Amazon users who have a record of completing these types of tasks. Each KW receives a small monetary reward from the requester for each task that they complete that is of a suitable standard to the requester. Amazon keeps a record of the performance of each KW and if desired, filters can be set by the requester, for example, permitting only KWs with a high success rate to perform the task. Each image classification task was published as one human intelligence task (HIT). For each HIT, KWs were given some background information and a written description of abnormal features of interest. (S1 Fig. is an example of the online questionnaire for each HIT) After reading through a descriptive illustration, KWs were asked if the test image had any suspicious features (thinning/notching of the neuroretinal rim or peri-papillary hemorrhage) which would warrant referral to an eye specialist. If none of the features were present, they were asked to designate the image as normal. There were no restrictions placed on the country of origin of workers. Any eligible worker could perform the task. Each image could be classified only once by each worker and there was no limit to how many images each worker could classify.

Based on previous estimations of repeated task accuracy in distributed human intelligence tasks, we requested 20 KW classifications per image.[6,7]

### Analysis

In order to assess the effect of categorization skill on classification accuracy we conducted two separate study designs:

1. No previous experience required—compensation 0.05cents (USD) per HIT
2. Previously completed ≥500 HITs with ≥90% approval—compensation 0.05cents per HIT

Both study designs were repeated to determine if the findings from trial 1 were reproducible. Using the selection of images as a pre-defined reference standard, we calculated the sensitivity and specificity for each of the study. This was calculated based upon the pooled responses of all image classifications (N = 2,540). In addition, we used a majority judgement method to identify the percentage of images correctly classified by the majority of KWs. We calculated a KW score determined by the ratio of votes for a normal or abnormal classification to the total number of votes for each classification. Receiver operating characteristic (ROC) curves were analysed for each study design and trial. The area under the ROC curves (AUC) were calculated as non parametric Mann-Whitney estimates and comparison between curves was performed using the z statistic for correlation. All analyses were performed using STATA v12.

## Results

All 2,540 classifications were obtained for 127 colour disc image (20 classifications per image) in under 24 hours. 54 images were designated as abnormal by pre-determined consensus, and 73 were designated normal. Table 1 highlights the baseline characteristics of the KWs for each trial. The mean time spent on each classification was under 1 minute. The time spent on each HIT did not differ significantly between correct and incorrect classification.

**Table 1:** Baseline characteristics of knowledge workers (KW) participation by study design for trials 1 and 2.

	Trial 1	
	0.05c	0.05c_500_90
**Number of different KWs**	78	63
**Mean(SD) number of HITs per KWs**	44(23)	34(19)
**Mean (SD) time on each HIT (secs)**	31(43)	40(50)
**Time to overall completion**	<24hrs	<24hrs
	**Trial 2**	
	0.05c	0.05c_500_90
**Number of different workers**	65	54
**Mean(SD) number of hits per KWs**	32(20)	28(14)
**Mean (SD) time on each hit (secs)**	25(32)	32(44)
**Time to overall completion**	<24hrs	<24hrs

(0.05c = study design 1—no previous experience; 0.05c_500_90% = study design 2—moderate experience)

**Fig 1:**
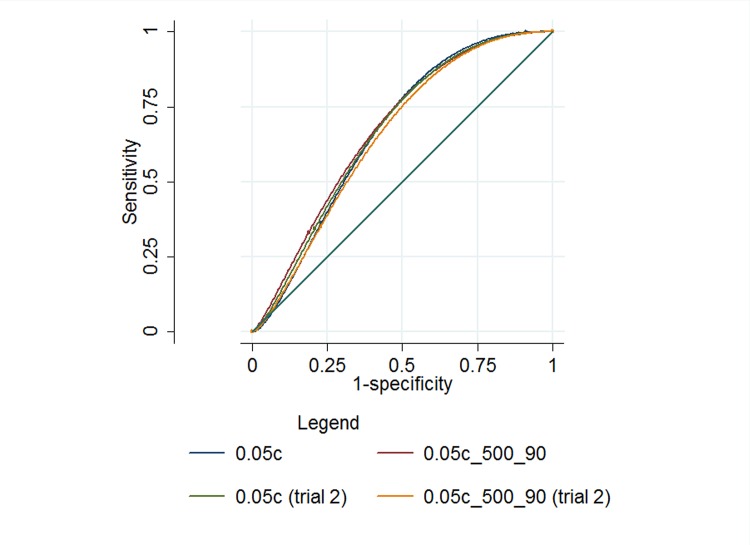
ROC curves for each study design in trials 1 and 2.

Table 2 shows the sensitivity and specificity of trials one and two. Fig. 1 illustrates the area under the ROC curve (AUC) for both study designs and trials. The sensitivity was between 83–88% across both trials, however the specificity was poor, ranging between 35–43%. There were no pairwise differences in the AUC between either trial or study design.

**Table 2:** The sensitivity, specificity and area under the ROC curve (AUC) for each study design in trials 1 and 2.

		Sensitivity	Specificity	AUC (95%CI)
**Trial 1**	0.05c	88.80%	35.50%	0.62(0.61–0.64)
	0.05c_500_90	83.98%	43.97%	0.64(0.62–0.66)
**Trial 2**	0.05c	86.20%	39.79%	0.63(0.61–0.65)
	0.05c_500_90	86.94%	36.10%	0.62(0.6–0.63)

(0.05c = study design 1—no previous experience; 0.05c_500_90% = study design 2—moderate experience)

**Fig 2:**
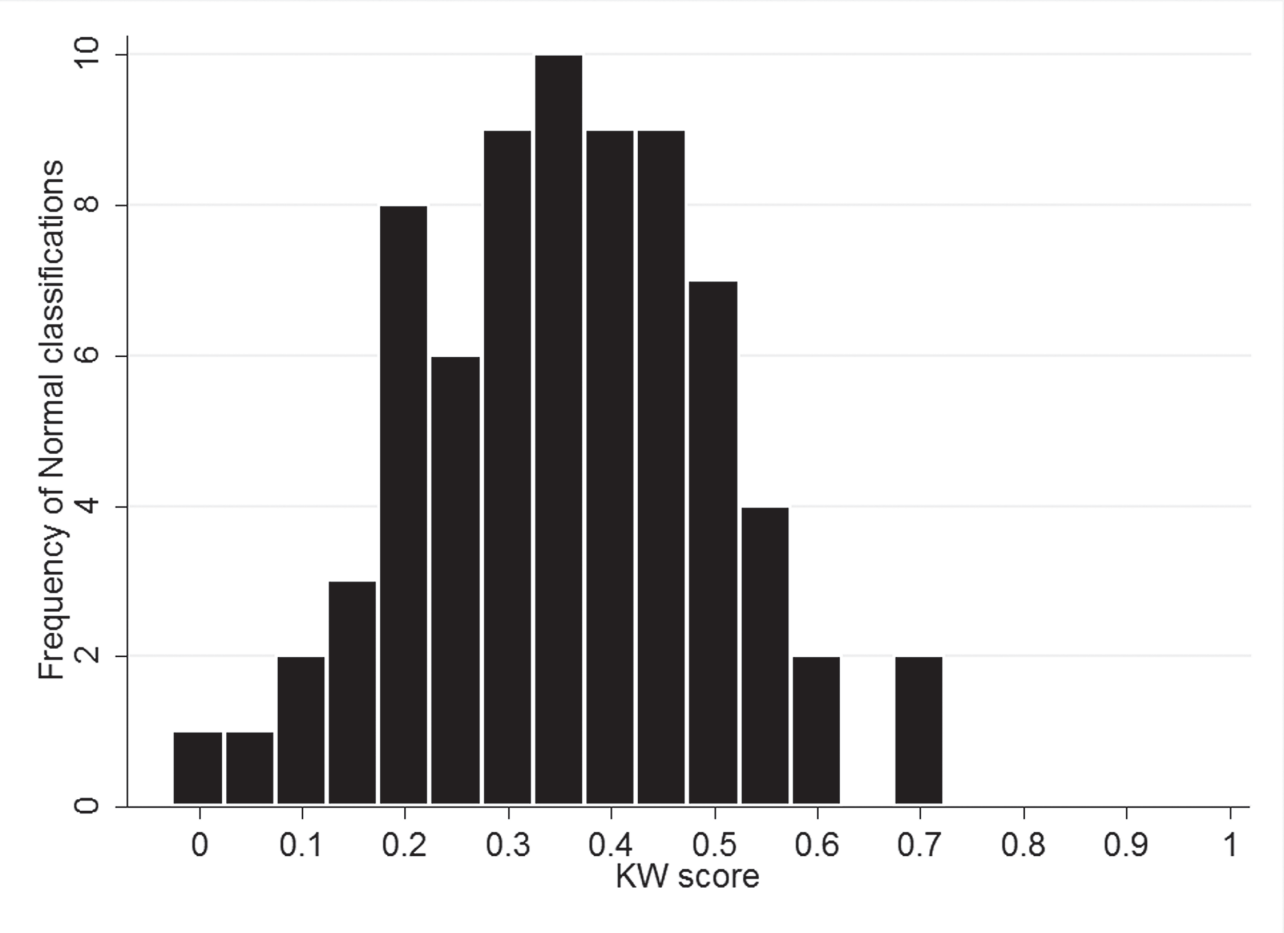
Histogram of classifications by KW score (calculated as ratio of votes for Normal to total number of votes for each classification) (N = 73) (0.05c trial 1).

**Fig 3:**
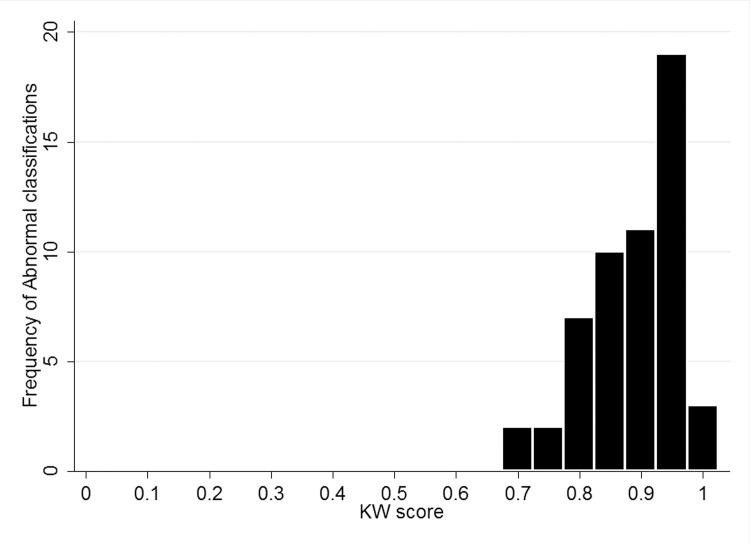
Histogram of classifications by KW score (calculated as ratio of votes for Abnormal to total number of votes for each classification) (N = 54) (0.05c trial 1).

Examining the percentage correctly classified (Table 3) shows that across both trials only between 8–36% of normal images were correctly assigned by the majority of KWs, whereas all abnormal images were correctly assigned by the majority of KWs. Figs. 2 and 3 show the classifications stratified by KW score for normal and abnormal images, demonstrating a much higher level of confidence in the true classification of abnormal.

**Table 3:** The percentage of Human Intelligence Tasks (HITs) correctly classified by the majority (>50%) of key workers (KW’s), with range of percentage of correct “votes” for each image category in brackets.

Trial 1	0.05c	0.05c_500_90
**Normal (N = 73)**	11%(0–70)	36%(0–90)
**Abnormal (N = 54)**	100%(70–100)	100%(65–100)
**Trial 2**	**0.05c**	**0.05c_500_90**
**Normal (N = 73)**	23%(0–70)	8%(5–60)
**Abnormal (N = 54)**	100%(60–100)	100%(65–100)

(0.05c = study design 1—no previous experience; 0.05c_500_90% = study design 2—moderate experience)

## Discussion

Crowdsourcing represents a compelling technique with potential for efficient analysis of medical images. Overall, we received 2,540 unique classifications of 127 images in several hours at minimal cost. In this study, we compared the accuracy of crowdsourcing in detecting disc abnormalities suggestive of glaucomatous optic neuropathy with the gold standard of senior image graders.

Overall, the area under the ROC curve (AUC) ranged between 0.62–0.64 for all study designs and trials conducted. This is lower than estimates of automated glaucoma detection from fundus images (0.88)[8] and from expert graders (0.86; 0.89–0.97).[4,9] Sensitivity/specificity estimates for expert binary grading of optic disc images was has been reported to vary between 76–78%/91–92%[10] with other reports suggesting an AUC of 0.80 for binary classification of optic disc images by general ophthalmologists.[11] However, is it recognized that subjective evaluation of the optic disc is a challenging task, often with poor agreement from graders.[12,13]. Using a simple online questionnaire, KWs were shown only 4 images for training, however a repeatable sensitivity of 83–88% was achieved. The principle limitation of the crowdsource in this task was the high rate of false positives due to the incorrect classification of normal images as abnormal resulting in a low specificity. Table 3 and Fig. 2 highlight that correct classification of abnormal images is performed with a much greater level of confidence by the crowdsource, compared to correct classification of normal images. Other variables involved in crowdsourcing, such as incentive, motivation and previous experience may also play a role in task accuracy, however based on our study designs we could not demonstrate a difference between moderately experienced and inexperienced MTurks users. In addition, as has been demonstrated previously[6,7], we also found that crowdsourcing is reliable and consistent, with minimal variation found between trials. Future studies of this technique should aim to more clearly define the range of acceptable normal features rather than focusing primarily on the detection of abnormal features and should aim to incorporate a structured training module.

This technique may find its primary utility in screening large Biobank datasets for more severe abnormalities, where grading time and physical infrastructure pose considerable limitations. Furthermore, a unique advantage of this technique may be to combine different imaging modalities to form part of a single classification, for example the crowdsource could be asked to classify a colour photograph and an OCT image of the same individual which may improve diagnostic precision. In summary, crowdsourcing is a novel tool in Ophthalmic image analysis that should be developed so that its full potential may be realised. Optimal crowdsourcing parameters such as incentivized rewards systems, better visualization methods, image presentation and expanded non-binary response options should be further explored so that their utility in improving the accuracy and reliability of this technique can be established.

## Supporting Information

S1 Data Raw data for analysis derived from Amazon MTurk. (ZIP)

S1 Fig An example of the online human intelligence task questionnaire presented to all key workers. (PDF)
