# Supplementary material for: Crowdsourcing as a Screening Tool to Detect Clinical Features of Glaucomatous Optic Neuropathy from Digital Photography
Source: PLoS One. 2015 Feb 18;10(2):e0117401. doi: 10.1371/journal.pone.0117401 (PMC4334897; doi:10.1371/journal.pone.0117401)
Supplement: S1 Fig — (PDF) [file pone.0117401.s002.pdf]

Select whether you would refer these images to an Ophthalmologist

This aim of this task is to classify which images of the optic nerve should be referred to an Ophthalmologist. You will be presented with an image of a human optic nerve. The optic nerve connects the eye to the brain and carries all the nerve impulses that provide vision, so it's an important structure! The optic nerve can be affected by many diseases, one of which is glaucoma. We want you to look at these images of optic nerves and help us decide which ones should be referred for a glaucoma check and which ones are normal.

Looking at an optic nerve can be difficult, with experts sometimes disagreeing! See the example of a normal optic nerve below. We want you to concentrate on the following features that can be affected in glaucoma.

1 - The neuroretinal rim : This is a rim of nerves between the central (often brighter in colour) cup of the optic nerve and the outer boundary of the nerve, the optic disc. The normal neuroretinal rim should have volume and should be round and even, much like a small tyre. If you see a round and even neuroretinal rim with no other features, these images should be classified as NORMAL. If you see a notch or thinning of the neuroretinal rim this could be a sign of early glaucoma and these images should be REFERRED. Look at the example images to see what a notch or thinning might look like.

2- Optic nerve hemorrhage : An optic nerve hemorrhage is a red streak near the edge of the nerve. It is a sign of glaucoma and may be associated with a notch or thinning of the neuroretinal rim. If you see any heamorrhages or red streaks around the optic nerve, these images should be REFERRED.

Have a look at the examples below, and keep in mind the features we want you to look out for.

Example images:

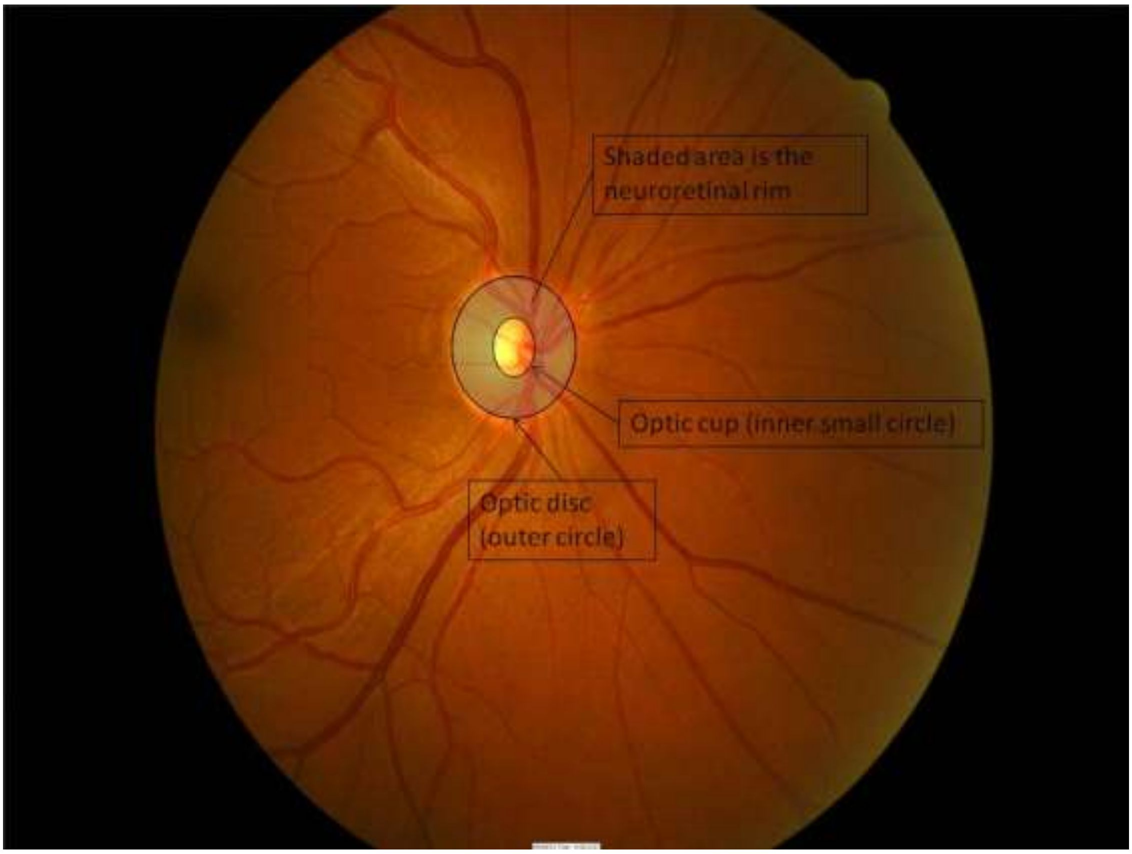

Normal

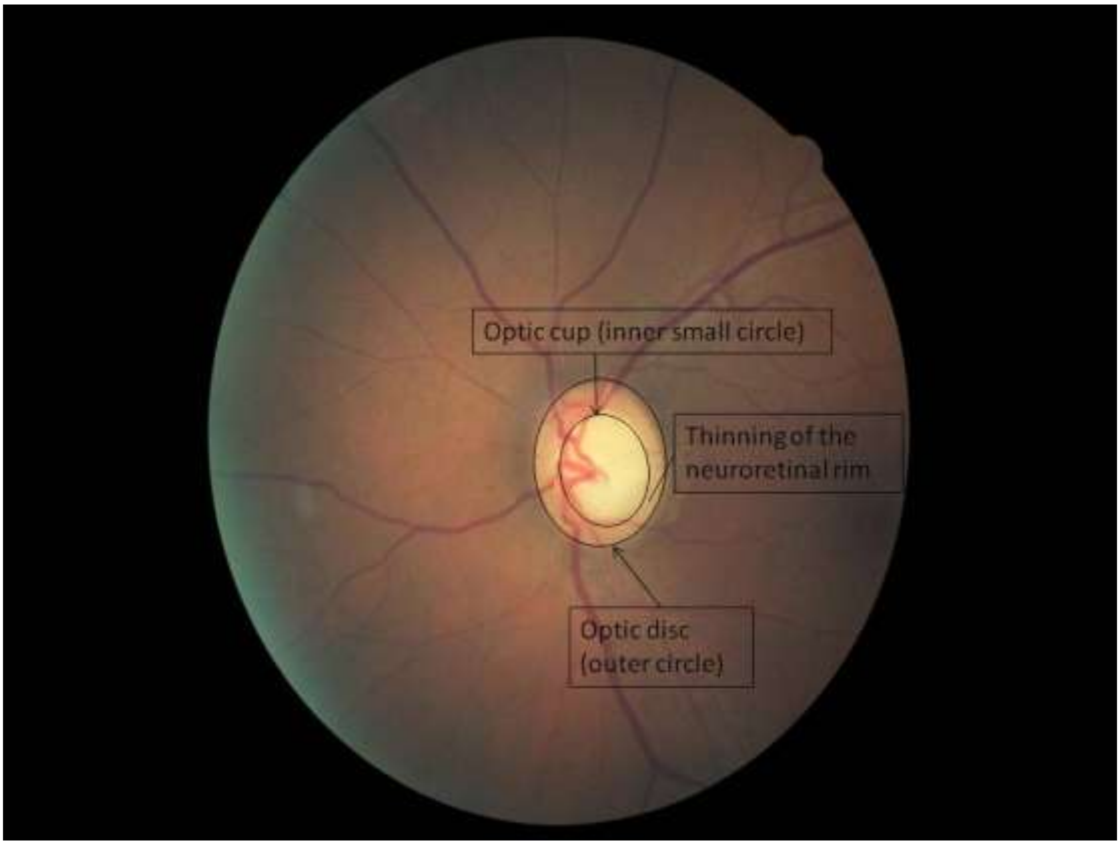

Thinning of the neuro-retinal rim - REFER

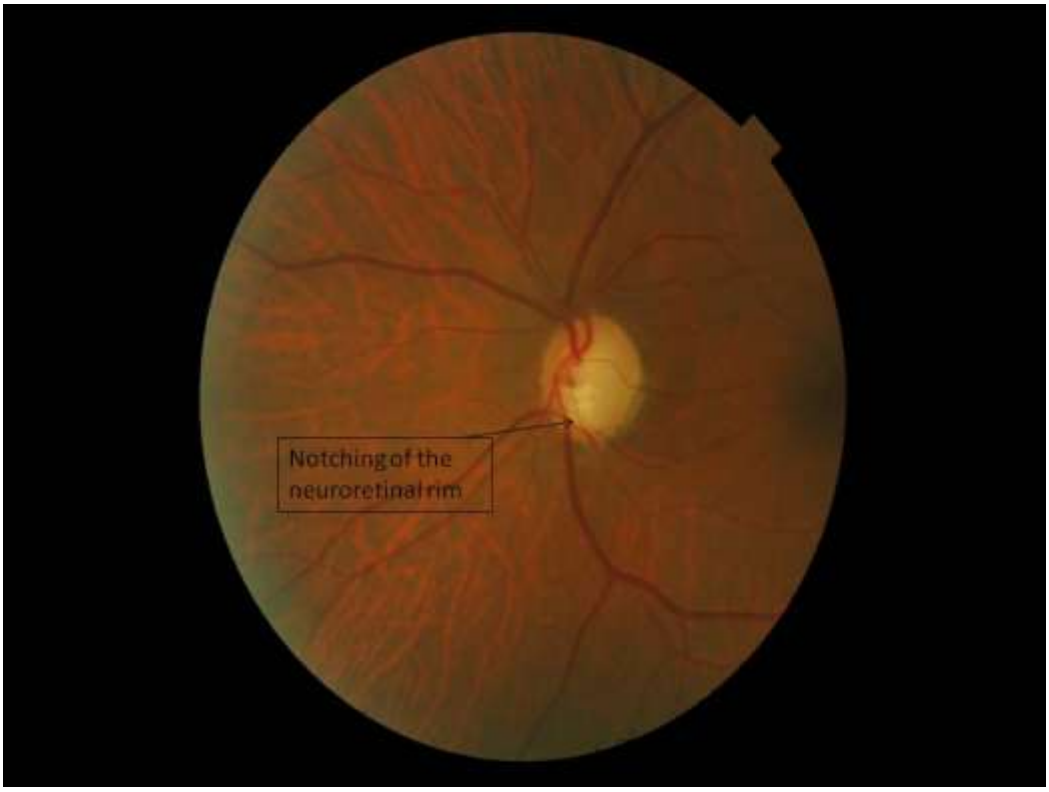

Notching of the neuro-retinal rim - REFER

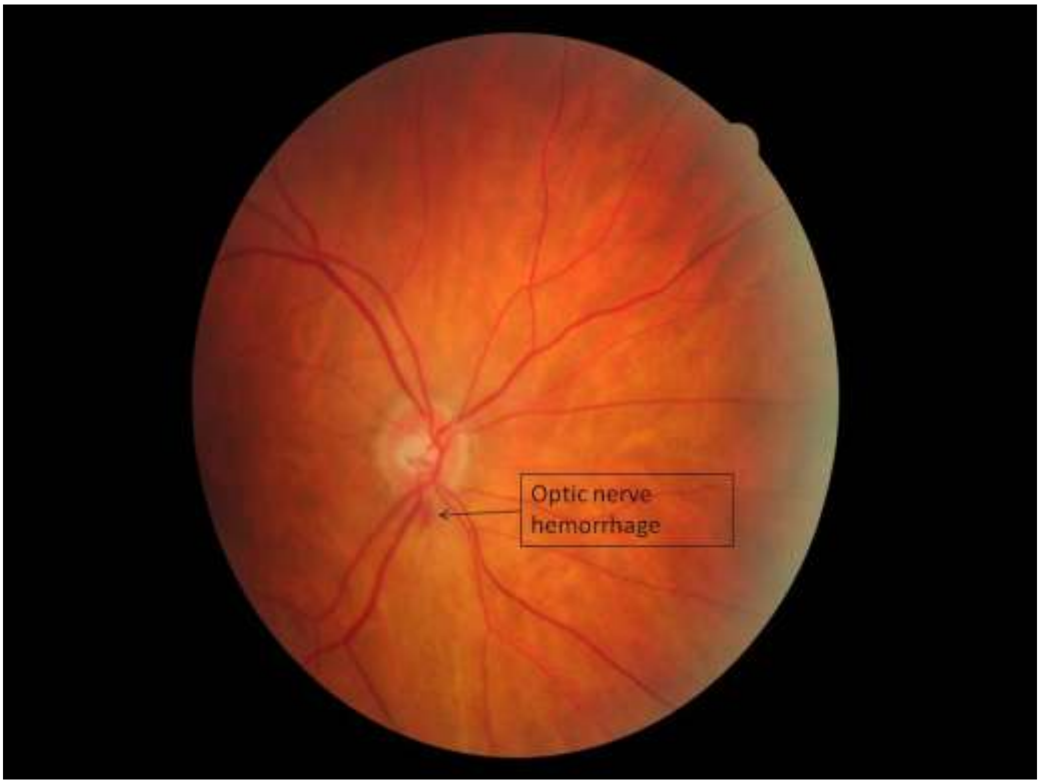

Optic nerve hemorrhage - REFER

END OF EXAMPLE IMAGES

Question:

Based on the above criteria, is this image normal or should it be referred to an Ophthalmologist for a glaucoma check?

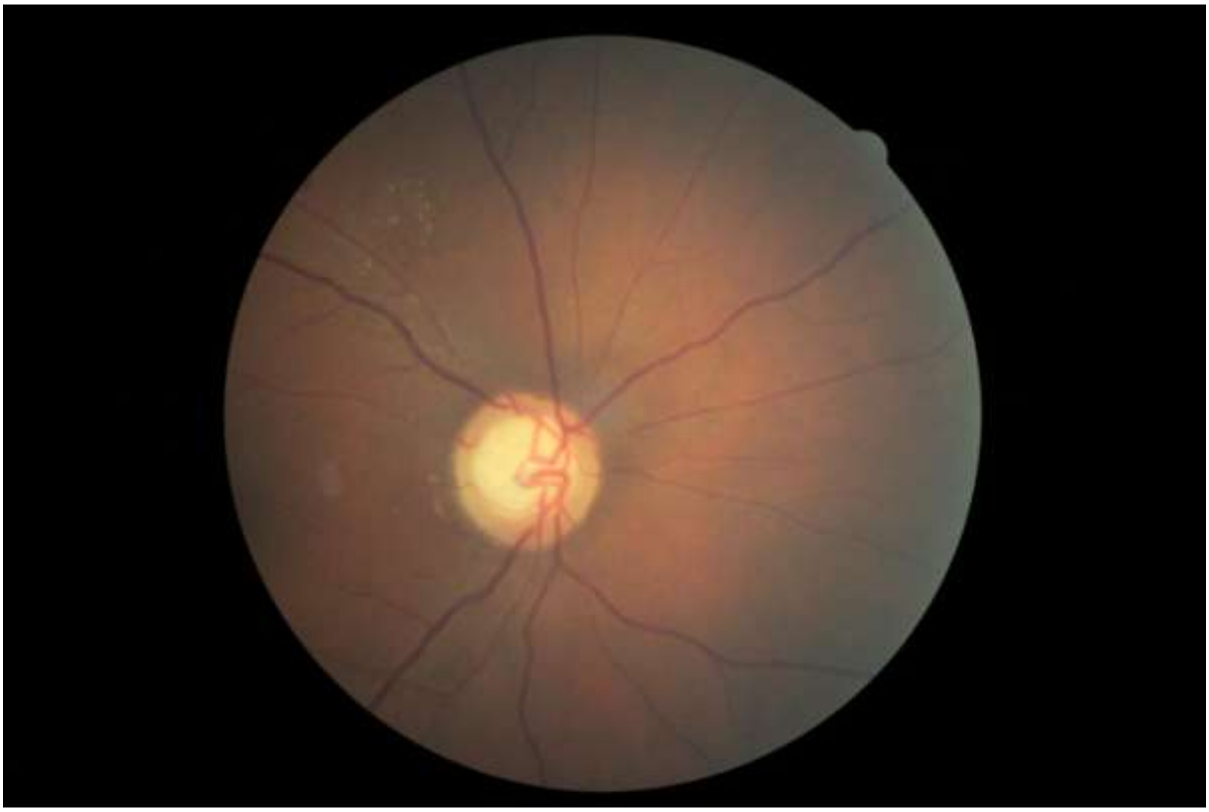

Please choose one answer:

□Normal

□REFER to an Ophthalmologist
